# Correlation study between facet joint cartilage and intervertebral discs in early lumbar vertebral degeneration using T2, T2* and T1ρ mapping

**DOI:** 10.1371/journal.pone.0178406

**Published:** 2017-06-01

**Authors:** Yi Zhang, Jianzhong Hu, Chunyue Duan, Ping Hu, Hongbin Lu, Xianjing Peng

**Affiliations:** 1 Department of Spine Surgery, Xiangya Hospital, Central South University, Changsha, Hunan, PR China; 2 Department of Radiology, Xiangya Hospital, Central South University, Changsha, Hunan, PR China; 3 Department of Sports Medicine and Research Center of Sports Medicine, Xiangya Hospital, Central South University, Changsha, Hunan, PR China; University of Umeå, SWEDEN

## Abstract

Recent advancements in magnetic resonance imaging have allowed for the early detection of biochemical changes in intervertebral discs and articular cartilage. Here, we assessed the feasibility of axial T2, T2* and T1ρ mapping of the lumbar facet joints (LFJs) to determine correlations between cartilage and intervertebral discs (IVDs) in early lumbar vertebral degeneration. We recruited 22 volunteers and examined 202 LFJs and 101 IVDs with morphological (sagittal and axial FSE T2-weighted imaging) and axial biochemical (T2, T2* and T1ρ mapping) sequences using a 3.0T MRI scanner. IVDs were graded using the Pfirrmann system. Mapping values of LFJs were recorded according to the degeneration grades of IVDs at the same level. The feasibility of T2, T2* and T1ρ in IVDs and LFJs were analyzed by comparing these mapping values across subjects with different rates of degeneration using Kruskal-Wallis tests. A Pearson’s correlation analysis was used to compare T2, T2* and T1ρ values of discs and LFJs. We found excellent reproducibility in the T2, T2* and T1ρ values for the nucleus pulposus (NP), anterior and posterior annulus fibrosus (PAF), and LFJ cartilage (intraclass correlation coefficients 0.806–0.955). T2, T2* and T1ρ mapping (all *P*<0.01) had good Pfirrmann grade performances in the NP with IVD degeneration. LFJ T2* values were significantly different between grades I and IV (*P*_L_ = 0.032, *P*_R_ = 0.026), as were T1ρ values between grades II and III (*P*_L_ = 0.002, *P*_R_ = 0.006) and grades III and IV (*P*_L_ = 0.006, *P*_R_ = 0.001). Correlations were moderately negative for T1ρ values between LFJ cartilage and NP (*r*_L_ = −0.574, *r*_R_ = −0.551), and between LFJ cartilage and PAF (*r*_L_ = −0.551, *r*_R_ = −0.499). T1ρ values of LFJ cartilage was weakly correlated with T2 (*r* = 0.007) and T2* (*r* = −0.158) values. Overall, we show that axial T1ρ effectively assesses early LFJ cartilage degeneration. Using T1ρ analysis, we propose a link between LFJ degeneration and IVD NP or PAF changes.

## Introduction

Low back pain (LBP) is a common disease that limits the activities of daily living, and is thus a major economic burden on health care systems. It is widely accepted that intervertebral lumbar discs (IVDs) and lumbar facet joints (LFJs) are the main sources of LBP[1, 2].

Degeneration changes in the IVD are characterized by a loss of hydrated proteoglycan-rich matrix of the nucleus pulposus (NP) and collagen, both of which affect the mechanics of the IVD[3]. In facet joint (FJ) osteoarthritis, patients demonstrate a loss of articular cartilage matrix, morphological reconstruction of the subchondral bone, and local tissue inflammation[2, 4, 5]. IVD degeneration may also change the mechanical integrity of the local lumbar spine, which may cause LFJ disorder, leading to FJ osteoarthritis[6]. Particularly in the early stages of IVD degeneration, it can be difficult to detect the small morphological changes of the LFJ on plain radiography, computed tomography, or conventional magnetic resonance imaging (MRI)[4]. In addition, morphological MRI grading systems for LFJ dysfunction (e.g., Weishaupt grading) tend to have poor inter-rater agreement[7].

With the improvements in MRI over the past two decades, new quantitative functional techniques-T2, T2* and T1ρ mapping, diffusion-weighted imaging, and delayed gadolinium-enhanced MRI have been used to reveal early biochemical changes in IVDs and in articular cartilage[8–11]. In IVDs and cartilage, T2 relaxation times are dependent on the quantity of water and the integrity of the proteoglycan(PG)-collagen matrix[12, 13]. During early disc degeneration, T2 mapping shows a decrease in water and glycosaminoglycan(GAG) content. When collagen degradation occurs in the LFJs, the increase in the water molecule content or an increased motion can be reflected as an increase of the T2 relaxation time, which indicates early damage[14]. In contrast, the T2* relaxation time reflects the “true” transverse relaxation time. Theoretically, T2* mapping and T2 mapping should similarly evaluate degeneration; however, some researchers report discrepant results for T2* values in degenerative articular cartilage as a result of different patterns of cartilage degeneration such as via fibrocartilage replacement or synovial fluid invasion[15]. Most recent studies have shown that T2* values decrease with an increase in cartilage degeneration, and these findings have suggested that T2* mapping could be used as a clinical diagnostic protocol for identifying cartilage and disc degeneration[15–18].

T1ρ mapping, however, uses a “spin lock” pulse to change the relaxation rates of water associated with large macromolecules, such as PGs and GAGs. Decreased GAG content may lead to an increased mobile proton density in bulk water and an increased in T1ρ relaxation times. Therefore, T1ρ mapping could be a reliable method for quantitatively assessing PG content in IVDs and cartilage without the need for a contrast agent[3, 19–21].

To the best of our knowledge, only a few articles have explored the relevance of biochemical parameters, such as T2 values for assessing LFJs and IVDs[7, 22]. The aims of this study were to explore whether axial T2, T2* and T1ρ mapping are feasible for evaluating early changes in LFJ, and to determine if there is an association of the T2, T2*, or T1ρ relaxation time between the LFJ and IVD in the degenerative process of the lumbar spine.

## Materials and methods

The study was approved by our institution’s research ethics committee of Xiangya Hospital, Central South University (NO. 201301008), and all volunteers involved in this study signed informed consent and agreed to the use of their imaging information for this analysis.

### Study subjects

This was a prospective study. A total of 22 volunteers, each with 5 lumbar IVDs and 10 LFJs, were recruited from September 2015 to March 2016. The inclusion criteria were healthy volunteers or other adults with nonspecific acute LBP, which was defined as pain lasting < 2 h per day over a period of < 3 months in subjects aged 20 to 45 years. The exclusion criteria were chronic LBP > 3 months, LBP caused by injury, LBP with a visual analog scale (VAS, 0–10 points, 0 stand for pain-free and gradually serious pain feeling with increasing points, 10 stand for the most serious) score ≥ 4 points, disc herniation or spinal stenosis, lumbar spondylolisthesis, tuberculosis, tumor or any other spinal diseases that would affect the stability of the lumbar spine and the loading state of the FJ. Subjects with a body mass index (BMI) > 25 or having other conditions that might generate unreadable data were also excluded from our study. All volunteers were examined by MRI in the morning (i.e., not after a long work day) to maintain the intervertebral discs in a relatively stable physiological situation.

### MR imaging protocol

All MR examinations were performed by a 3.0 T MR unit (Signa HDxt; GE Medical Systems, Waukesha, WI, USA) and a dedicated eight-channel spine coil. Morphological (sagittal and axial FSE T2-weighted imaging) and biochemical (axial T2, T2* and T1ρ mapping) sequences were performed, covering the IVDs L1/2 to L5/S1.

T2 and T2* mapping were carried out in the axial plane using a 2D multi-echo spin-echo (MESE) sequence and 2D multi-echo fast spoiled gradient-echo (ME-FSGRE) sequence, respectively (S1 Text). Five axial slabs with three slices each covered the five lumbar segments (discs and FJs L1/2 to L5/S1) in each patient for both techniques. Each two-dimensional slab (group of three slices) was aligned along the corresponding intervertebral disc. The central slice (slice 2 of 3), which covered the intervertebral disc and the slice caudal to the central slice (slice 3 of 3) were used for T2 and T2* evaluation of the IVDs and the FJs respectively.

T1ρ mapping was performed in the axial plane by 3D spoiled gradient-echo (SPGR) sequence (S1 Text). The spin-lock frequency was set to 500 Hz and the spin-lock times (TSLs) of 0, 10, 40 and 80 ms were used for acquisition. Each three-dimensional slab (group of eight slices) was aligned along the corresponding intervertebral disc. The slice (slice 5 of 8) covering the intervertebral disc and the slice caudal to central slice (slice 6 of 8) were used for T1ρ evaluation of the IVDs and the FJs respectively. All sequences and sequence parameters are shown in Table 1.

**Table 1:** Parameters of the morphological and biochemical MRI sequences.

Sequences Parameter	T2WI Sagittal	T2WI Axial	T2 mapping Axial	T2* mapping Axial	T1ρ mapping Axial
**Pulse sequence**	FSE	FSE	2D MESE	2D MEFSGRE	3D SPGR
**Repetition time (ms)**	2900	5080	1000	450	7.5
**Echo time or TSLs (ms)**	120	94	12.2; 24.4; 36.6; 48.9; 61.1; 73.3; 85.5; 97.7	5.1; 10.6; 16.1; 21.6; 27.1; 32.6; 38.1; 43.6	0; 10; 40; 80
**Flip angle (°)**	-	-	-	30	60
**Field of view (mm)**	320×320	180×180	180×180	180×180	140×140
**Matrix**	416×320	320×320	256×256	256×256	256×128
**Slice thickness (mm)**	4	3	4	4	3
**Interslice gap (mm)**	1	0.3	2	2	-
**Number of slices/slab**	11	8/5	3/5	3/5	8/5
**Acquisition time (min: s)**	01:27	06:52	04:34	07:48	03:19

### Image analysis

Two experienced readers independently assessed the morphology of the IVDs from L1/2 to L5/S1 according to the Pfirrmann grading system on sagittal T2 FSE images. The Pfirrmann grading system is primarily based on changes in signal intensity from the NP, the distinction between the NP and the annulus fibrosus, and disc height[23–25]. The distribution of the 101 IVDs with respect to the Pfirrmann grades was determined independently by an experienced musculoskeletal radiologist (XJP) with 15 years’ experience, and a spinal surgeon (CYD) with 20 years’ experience. A senior radiologist with 13 years’ experience manually selected the regions of interest (ROIs) on the working station (Function Software, ADW 4.4 workstation, GE Medical Systems, Waukesha, WI, USA) to measure the mapping values (S1 Fig). The ROIs for the IVDs were drawn on T2, T2* and T1ρ maps according to the subject’s anatomic shape. In addition, one large, circular ROI was selected for the NP, and two smaller circular ROIs were selected for the anterior annulus fibrosus (AAF) and the posterior annulus fibrosus (PAF), respectively. The ROI of the NP in the mid-sagittal line was about 50% to 60% of the disc diameter. The ROIs of the LFJs were drawn on each map across both articular surfaces of each FJ. All ROIs were selected and calculated on the left and right sides.

### Statistical analysis

Mean values were used for statistical analyses. The intraclass correlation coefficients (ICC) with 95% confidence intervals (95% CIs) were used to evaluate the reproducibility of T2, T2* and T1ρ relaxation times, and an ICC>0.75 was considered excellent agreement. The Kruskal-Wallis and Nemenyi tests were used to compare the differences in the T2, T2* and T1ρ values of discs and LFJs among the different Pfirrmann grades. A Pearson correlation analysis was performed to compare T2, T2* and T1ρ values of the disc and LFJs. The absolute value of correlation coefficients *r* were used to indicate very strong correlation (*r* = 0.80 to 1.00), strong correlation (*r* = 0.60 to 0.79), moderate correlation (*r* = 0.40 to 0.59), weak correlation (*r* = 0.20 to 0.39) or no correlation (*r*<0.20). All statistical evaluations were performed using PASW Statistics software (19.0, SPSS, Chicago, IL, USA). A *P*< 0.05 was considered statistically significant.

## Results

### Volunteers characteristics

A total of 22 volunteers were recruited (12 women and 10 men; mean age, 33.2 years, range, 21 to 44 years). In all, 202 LFJs and 101 IVDs from L1/2 to L5/S1 were assessed. The morphological IVD Pfirrmann grades on sagittal T2WI are shown in S1 Fig. The reproducibility for the three methods was excellent and the data are presented in Table 2.

**Table 2:** The interclass correlation coefficients (ICC) with 95% confidence intervals (CIs) of T2, T2* and T1ρ mapping.

ROIs	T2 mapping		T2* mapping		T1ρ mapping	
	ICC	95% CI	ICC	95% CI	ICC	95% CI
**NP**	0.908	0.870–0.928	0.899	0.854–0.922	0.892	0.849–0.916
**AAF**	0.895	0.850–0.920	0.931	0.882–0.941	0.942	0.926–0.951
**PAF**	0.941	0.925–0.951	0.955	0.942–0.967	0.951	0.938–0.964
**LFJ**	0.807	0.785–0.820	0.831	0.808–0.847	0.825	0.805–0.842

Abbreviations: ROI, regions of interest; NP, nucleus pulposus; AAF, anterior annulus fibrosus; PAF, posterior annulus fibrosus; LFJ, lumbar facet joint.

#### Differences in the T2, T2* and T1ρ values for various anatomic areas of the IVDs among the four Pfirrmann grades

**Fig 1:**
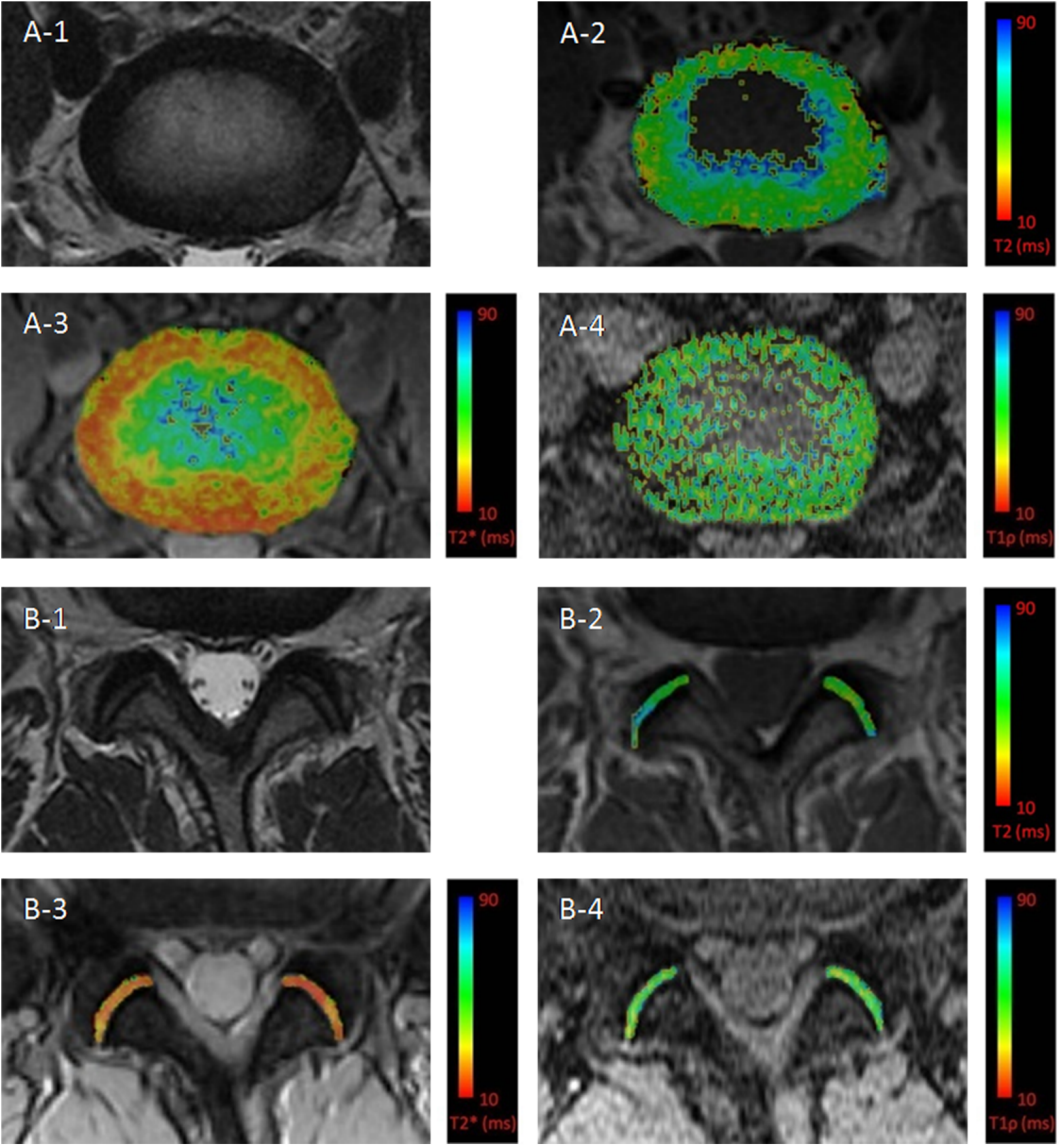
Illustration of the intervertebral discs (IVDs) and lumbar facet joints (LFJs) in morphological sequence and T2/T2*/T1ρ mapping in one volunteer. (A-1) Morphology of the IVD in the FSE sequence with Pfirrmann grade I. (A-2 to A-4) Color-coded T2/T2*/T1ρ map of the L3/4 IVD, respectively. (B-1) Morphology of the LFJ of the same segment adjacent to the L3/4 IVD. (B-2 to B-4) Color-coded T2 /T2*/T1ρ map of L3/4 LFJ respectively.

**Fig 2:**
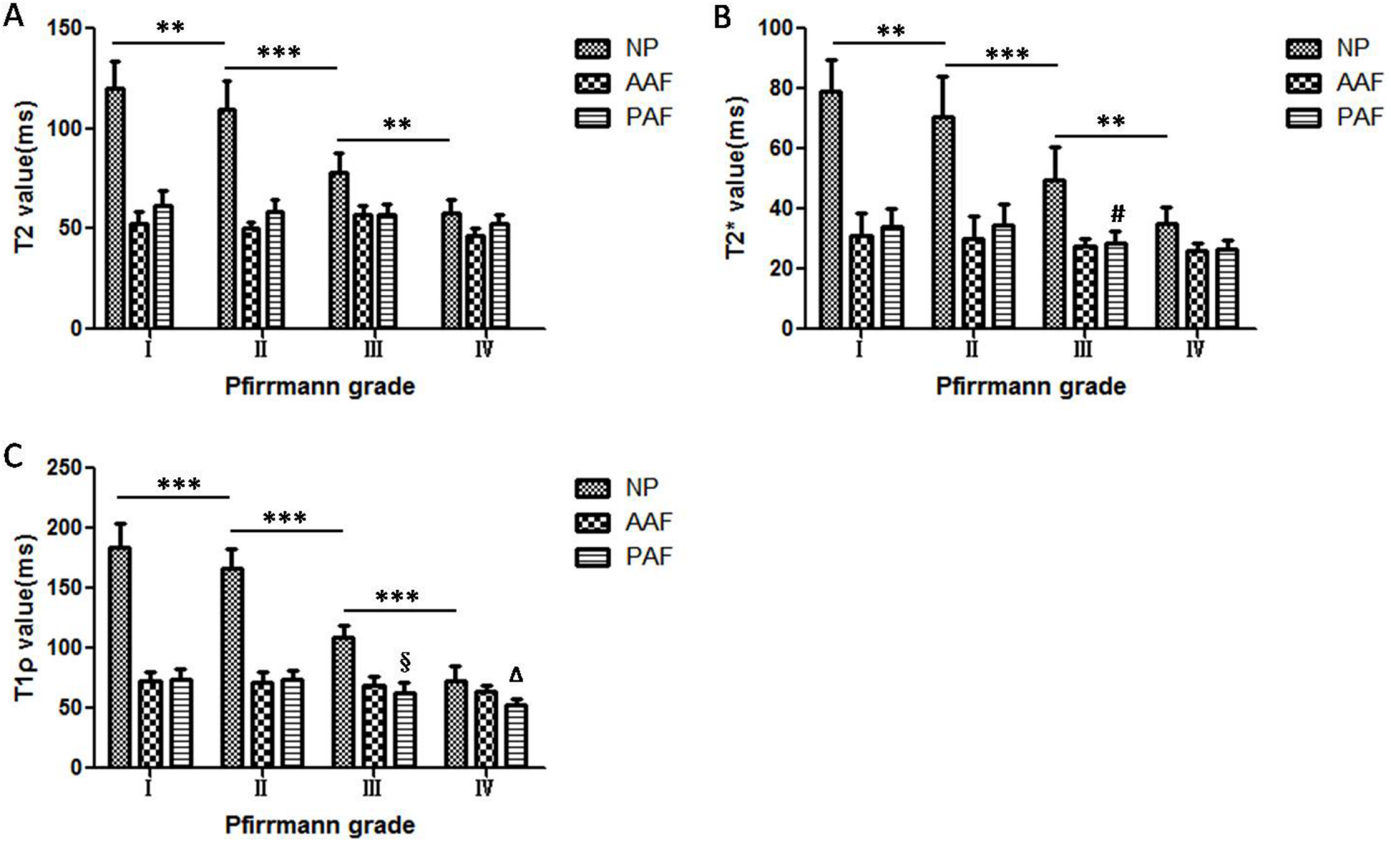
Changes in T2, T2* and T1ρ values of intervertebral discs (IVDs) with increasing Pfirrmann grade. Changes in the (A) T2, (B) T2* and (C) T1ρ values of IVD with degeneration grade. *P<0.05; **P<0.01; ***P<0.001; ^#^difference between Pfirrmann grade II and III in PAF; ^§^difference between grade II and III in PAF; ^Δ^difference between grade III and IV in PAF. No disc corresponding to Pfirrmann grade V was found in this study.

The images of the axial morphological sequence (FSE) and the T2, T2*, and T1ρ mapping of the IVD and LFJ are shown in Fig 1. The T2, T2* and T1ρ values of the NP, AAF and PAF with increasing Pfirrmann grade are shown in Fig 2 and Table 3. There were statistically significant differences in the T2, T2* and T1ρ values for the NP among the four grades (all *P*<0.001), especially in the T1ρ values between grades II (166.93±15.87 ms) and III (108.98±10.31 ms) (*P* = 0.000). There were no significant differences in the T2, T2* and T1ρ values for the AAF (all *P*>0.05). Although there were differences in the T2, T2* and T1ρ values for the PAF (Fig 2), we found no significant differences in the T2 values among the grades (*P* = 0.051, Fig 2A). However, a significant difference was found in the T2* values between Pfirrmann grades II (34.34±7.28 ms) and III (28.79±4.04 ms) (*P* = 0.005, Fig 2B) and in the T1ρ values between Pfirrmann grades II (73.67±7.55 ms) and III (63.24±7.85 ms) (*P* = 0.000, Fig 2C) and grades III and IV (52.69±4.99 ms) (*P* = 0.006, Fig 2C).

**Table 3:** T2, T2* and T1ρ values and the distribution of IVD and LFJ.

	T2 values (ms)				T2* values (ms)				T1ρ values (ms)			
	I	II	III	IV	I	II	III	IV	I	II	III	IV
**NP**	119.80±14.12	109.55±14.57	78.05±9.82	58.22±6.10	79.16±10.22	70.36±13.54	49.46±11.12	35.22±5.52	183.90±20.30	166.93±15.87	108.98±10.31	73.20±12.39
**AAF**	52.55±5.74	50.29±3.43	49.39±4.68	46.61±3.52	30.97±7.51	30.31±7.28	27.67±2.33	25.99±2.50	72.31±7.68	71.59±8.39	68.62±7.41	63.82±5.08
**PAF**	61.89±7.35	58.77±5.80	56.97±5.45	52.94±4.09	34.15±5.82	34.34±7.28	28.79±4.04	26.59±2.90	74.15±8.92	73.67±7.55	63.24±7.85	52.69±4.99
**L LFJ**	57.17±6.00	57.62±6.23	57.01±5.03	58.15±2.68	22.02±3.48	20.62±3.00	20.01±3.11	19.33±3.01	49.37±4.67	50.70±4.38	55.08±3.69	60.80±3.65
**R LFJ**	57.44±5.33	57.67±7.13	57.15±7.18	59.53±5.31	21.89±3.17	20.78±3.35	20.19±3.16	19.21±2.88	49.49±3.75	50.71±4.36	54.37±3.18	60.46±4.32

Abbreviations: IVD, intervertebral discs; LFJ, lumbar facet joint; NP, nucleus pulposus; AAF, anterior annulus fibrosus; PAF, posterior annulus fibrosus.

#### Correlations of T2, T2* and T1ρ values between the LFJ cartilage and IVD

**Fig 3:**
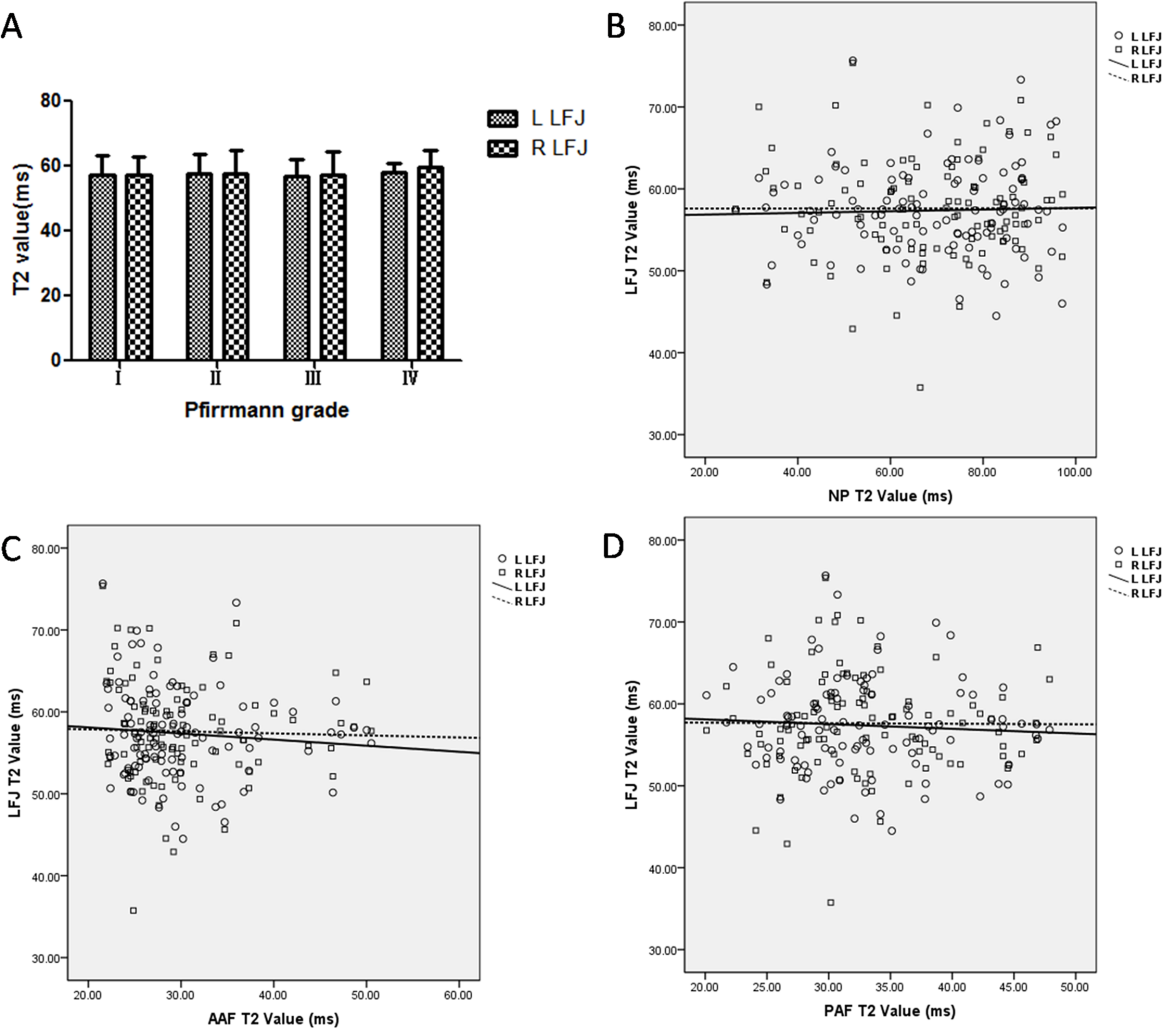
Changes in the T2 values of the lumbar facet joint (LFJ) with changes in the intervertebral discs (IVDs). (A) Changes in the T2 values of the LFJ with degeneration grade; (B-D) Correlations between the T2 values of the left/right LFJ cartilage and (B) the nucleus pulposus (NP), (C) the anterior annulus fibrosus (AAF), and (D) the posterior annulus fibrosus (PAF).

**Fig 4:**
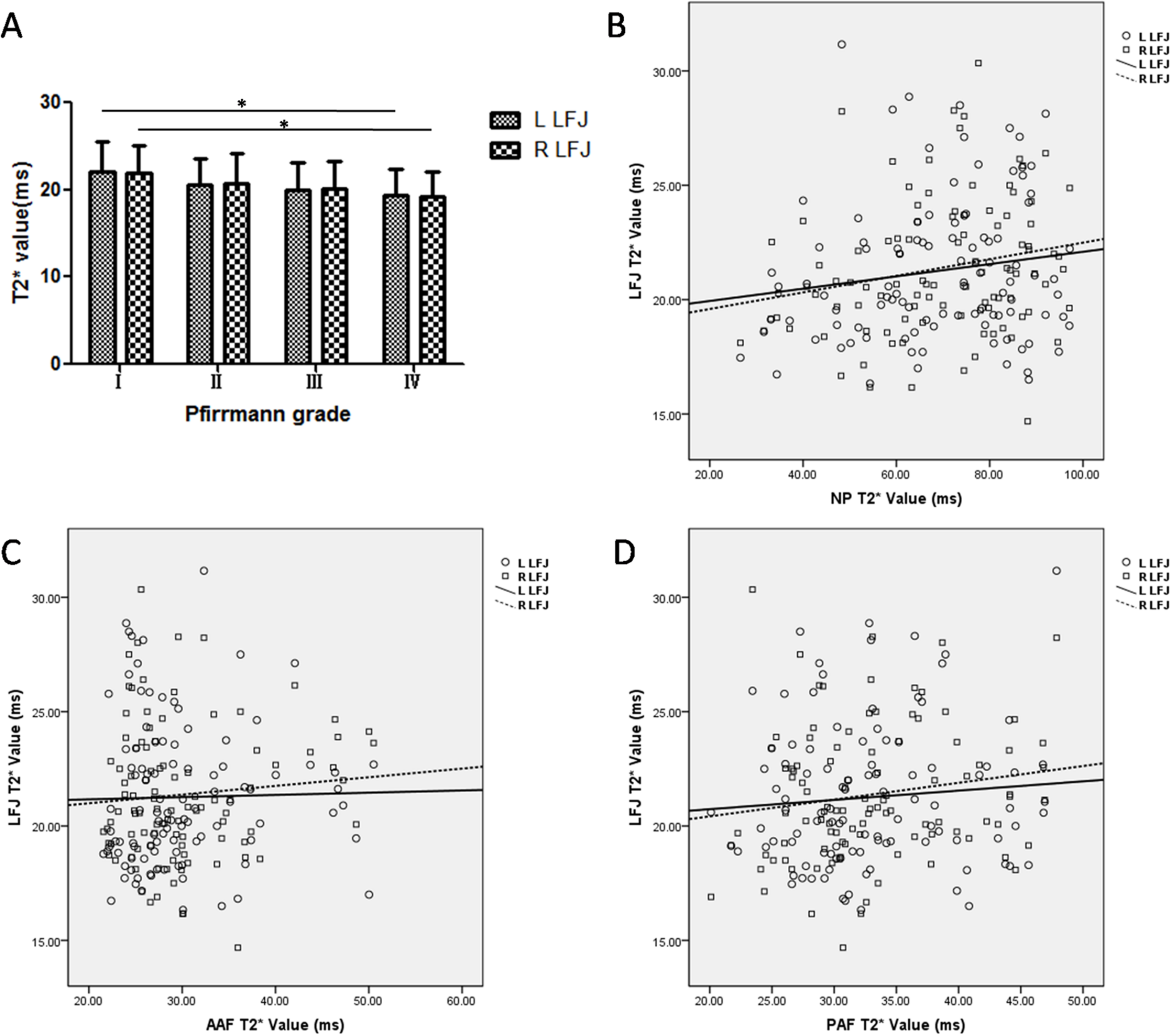
Changes in the T2* values of the lumbar facet joint (LFJ) with changes in the intervertebral discs (IVDs). (A) Changes in the T2* values of the LFJ with degeneration grade. (B–D) Correlations between the T2* values of the left/right LFJ cartilage and (B) the nucleus pulposus (NP), (C) the anterior annulus fibrosus (AAF), and (D) the posterior annulus fibrosus (PAF). *P <0.05.

**Fig 5:**
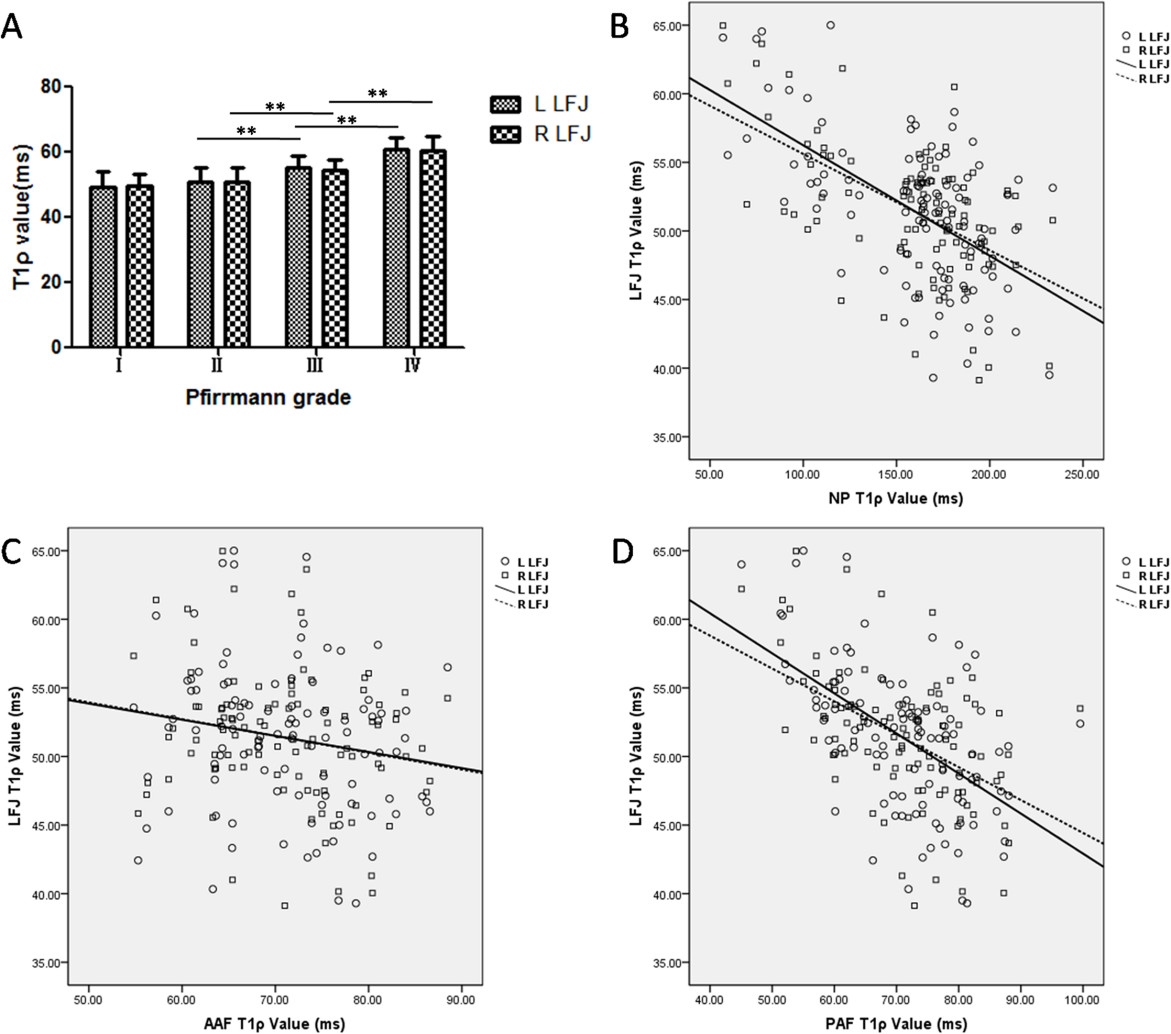
Changes in the T1ρ values of the lumbar facet joint (LFJ) with changes in the intervertebral discs (IVDs). (A) Changes in the T1ρ values of the LFJ with degeneration grade. (B–D) Correlations between the T1ρ values of the left/right LFJ cartilage and (B) the nucleus pulposus (NP), (C) the anterior annulus fibrosus (AAF), and (D) the posterior annulus fibrosus (PAF). **P <0.01.

The T2, T2* and T1ρ values for the LFJ cartilage tended to change with an increase in Pfirrmann grade (Figs 3A, 4A and 5A and Table 3). The correlations between the T2, T2* and T1ρ values for the LFJ and the IVD are shown in Figs 3, 4 and 5. For the T2 value of the LFJ, no significant differences were found among the four groups (*P*_*L*_ = 0.961, *P*_*R*_ = 0.855, Fig 3A). The T2 value also showed no correlation between the LFJ cartilage and the NP (*r*_L_ = 0.081,*r*_R_ = 0.033, Fig 3B), the AAF (*r*_L_ = −0.169,*r*_R_ = 0.046, Fig 3C), or the PAF (*r*_L_ = −0.004,*r*_R_ = 0.047, Fig 3D).

The T2* values for the LFJs showed a significant difference among the four Pfirrmann grade groups and between grades I (left [L],22.02±3.48 ms; right [R], 21.89±3.17 ms) and IV (L, 19.33±3.01 ms; R,19.21±2.88 ms) (*P*_L_ = 0.032, *P*_R_ = 0.026, Fig 4A). The NP T2* values were weakly correlated with the LFJ T2* value (*r*_L_ = 0.148,*r*_R_ = 0.215, Fig 4B). Neither the AAF T2* value (*r*_L_ = 0.021, *r*_R_ = 0.087) nor the PAF T2* value (*r*_L_ = 0.082,*r*_R_ = 0.160) was correlated with that of the LFJ (Fig 4C and 4D).

Fig 5A shows the mean T1ρ values for the LFJ at different IVD Pfirrmann grades. There were statistically significant differences among the four groups and between grades II (L, 50.70±4.38 ms; R, 50.71±4.36 ms) and III (L, 55.08±3.69 ms; R, 54.37±3.18 ms) (*P*_L_ = 0.002, *P*_R_ = 0.006), and grades III and IV (L, 60.80±3.65 ms; R, 60.46±4.32 ms) (*P*_L_ = 0.006, *P*_R_ = 0.001). A moderately negative correlation was found between the LFJ and the NP (*r*_L_ = −0.574,*r*_R_ = −0.551, Fig 5B) for the T1ρ value, as well as between the LFJ and the PAF (*r*_L_ = −0.551,*r*_R_ = −0.499, Fig 5D). There was no correlation between the LFJ and the AAF (*r*_L_ = −0.173, *r*_R_ = −0.200, Fig 5C).

#### Correlations between T1ρ values and the T2 and T2* values of the LFJ

**Fig 6:**
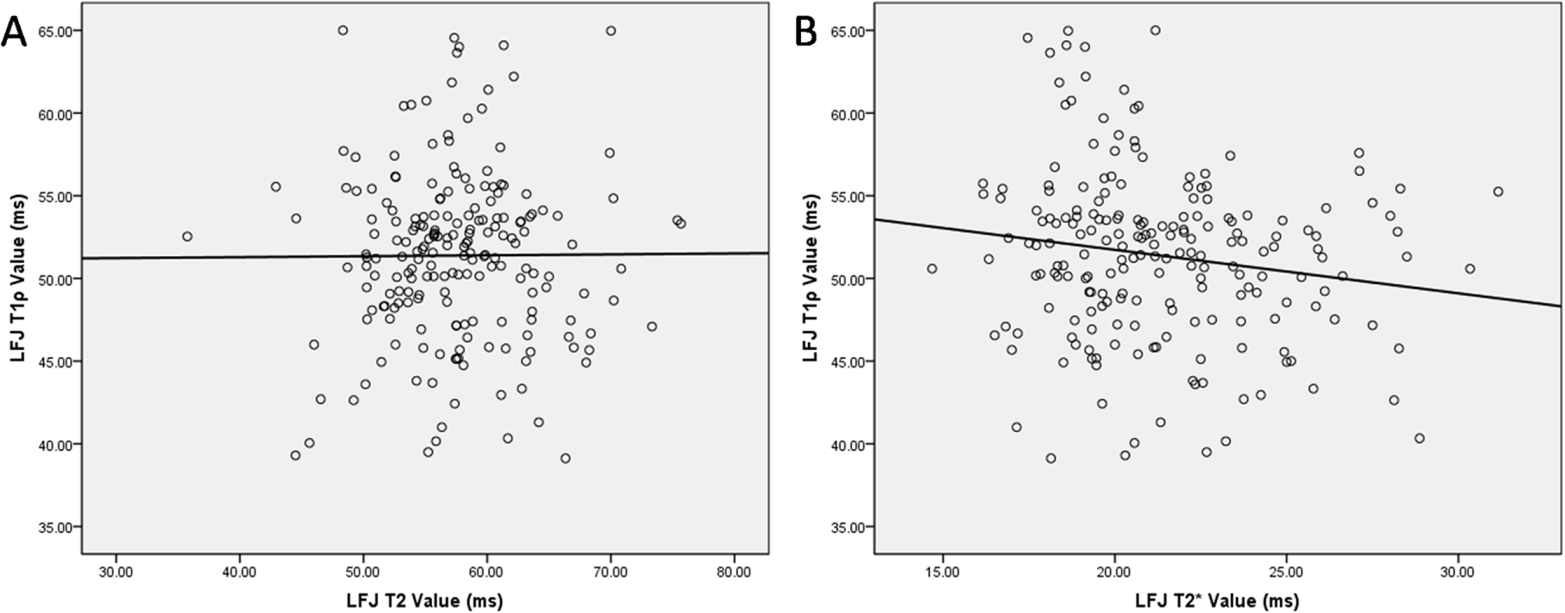
Correlation between T1ρ and T2 or T2* values of the lumbar facet joints (LFJs).

To avoid the mutual influence of the different methods, we next explored the relationships among the T1ρ value and the other two methods. Our results showed that the T1ρ values for LFJ were weakly correlated with the T2 (*r* = 0.007) and T2* values (*r* = −0.158) (Fig 6).

## Discussion

Quantitative MRI has attracted increasing attention over the past two decades as a way to evaluate articular cartilage and IVD[18, 26–29]. Many authors believe that T2, T2* or T1ρ mapping are reliable for investigating early degeneration of cartilage[13, 15, 20, 30]. To the best of our knowledge, little research has been done to determine the T2, T2* and T1ρ relaxation times of the LFJ and correlate changes in relaxation times of the IVD with degenerative changes.

Previous studies have reported the ranges of T2, T2* and T1ρ values for healthy and osteoarthritis (OA) cartilage in knees[31–34]. We performed axial T2, T2* and T1ρ mapping of the LFJ and the IVD (Table 3); the values of the LFJ are close to those for knee joint cartilage[7, 22]. The mean and range of T1ρ values for the FJ cartilage in our study are the first such measurements to be reported. T2, T2* and T1ρ mapping performed well in evaluating the degeneration changes of the IVDs in our study, with significant differences noted for the T2, T2* and T1ρ values for the NP between Pfirrmann grades, especially between grades II and III. Furthermore, we measured a significant difference in the T2* values for the PAF between Pfirrmann grades II and III, as well as in the T1ρ values for the PAF between grades II and III and grades III and IV; these results are similar to those reported by others[3, 18].

The changes in the molecular biochemical components in degenerated IVDs can be accurately evaluated by T2, T2* and T1ρ mapping. T2 mapping might be more sensitive to tissue hydration; however, T2* mapping might be more sensitive to collagen integrity, and T1ρ mapping can show slow-motion interactions between macromolecules and bulk water in the extracellular matrix[7, 18, 21, 26, 28]. The main degenerative changes to the IVD are the loss of PG/GAG and dehydration[22, 23, 25]. Considering the load-bearing characteristics of IVDs, the NP and PAF may suffer more stress than the AAF and show earlier and more severe signs of degenerative change. In previous work, the T1ρ relaxation time was shown to be associated with the change of PG/GAG, and T1ρ mapping could also be used to detect early changes of PG/GAG composition in IVD[26, 27]. Here, we found that T1ρ mapping was more sensitive in detecting early changes in the IVD than T2 and T2* mapping.

In this study, we noted different sensibilities for the T2, T2* and T1ρ values of the FJ in detecting degeneration of the LFJ with increasing Pfirrmann grades of the IVD. T2* and T1ρ values could detect LFJ changes with increasing Pfirrmann grades of IVD, but no significant results were found for T2 values. Stelzeneder D et al. reported comparable results regarding FJ T2 values in segments with normal and abnormal Pfirrmann scores[7]. Compared with T2 and T2*, T1ρ values showed better sensitivity for detecting early degenerative changes in the FJ cartilage. In the LFJ, the changes in degeneration as measured by T2, T2* and T1ρ values were similar to previous results found in other joints[12, 31, 32, 35, 36]. LFJ cartilage degeneration is mainly associated with alterations in water, collagen and PG, which play important roles in different stages of degeneration[5, 8]. Previous studies in other joints have shown that at a very early stage of cartilage degeneration, the GAG content might change more obviously than the collagen content[10, 19], and this change could be detected by the T1ρ relaxation time. Therefore, in LFJ degeneration, T1ρ values seem to offer a more sensitive and reliable method to assess the early changes in the cartilage.

Another meaningful finding in this study was that the degenerative changes of the LFJ may correlate with IVD degeneration, as measured through T1ρ values, especially the NP and the PAF. We did not find any meaningful correlations for T2 and T2* values for the LFJ. A previous study found that FJs were weakly correlated with T2 values for the PAF according to axial T2 mapping[7]. However, there is a lack of more direct studies that could prove the correlation between LFJ degeneration and the changes in the IVD measured using other mapping values. As described in the literature, changes in the lumbar spine mechanics environment during IVD degeneration may affect the loading distribution on the subchondral bone of the LFJ, and this would lead to functional and morphological changes in the cartilage[4, 37]. Changes in the biochemical values that reflect early degeneration of the IVD are dependent on interactions between free water and matrix molecules. T1ρ values that reflect dehydration and loss of the PG in the NP were negatively correlated with that of the LFJ, suggestive of increased water content and decreased GAG content of the LFJ cartilage. Degenerative discs can cause an increase in force transmission across the FJs and, in turn, a change in the orientation and stability of the FJ can affect the disc. Degeneration in the PAF causes a reduction of load-bearing capacity, which leads to degeneration of the LFJ cartilage and, later, a further change in the PAF. Our results also suggest that the NP and the PAF degenerate before the AAF, and that PAF degeneration is closely associated with FJOA. In this study, all of the volunteers who were recruited were less than 45 years old and the Pfirrmann grades of the IVDs for these subjects were mainly distributed within grades I to III. The biochemical changes in the LFJ in our study cohort were mainly due to the loss of PG/GAG content, as described before. Low GAG levels are represented by high T1ρ values, and the loss of PG/GAG results in an increase in the T1ρ relaxation time in articular cartilage[20]. However, in degenerated IVDs, the reduced water content is recognized as a contributing factor that causes a decrease in T1ρ values[27, 38]. Therefore, we found a negative correlation between LFJs and IVDs confirmed only by T1ρ values. T1ρ values for the LFJ were weakly correlated with the T2 and T2* values, suggesting that changes to the LFJs are detected earlier by T1ρ, and that the sensitivity of T1ρ exceeds that of T2* and T2 measurements.

The major limitation of our study was that there was no histological assessment of disc degeneration or LFJ cartilage. We should also point out that the T2, T2* and T1ρ values of the LFJ cartilage do not remove the effect of joint fluid, and this may be a result of relatively poor resolution of the mapping. However, there appears to have been a limited effect of joint fluid on our results.

Our study uniquely assessed the feasibility of detecting IVD and LFJ degeneration using three quantitative biochemical methods on axial scanning. Our results demonstrated that T1ρ values are more sensitive than T2 and T2* values for assessing the early degenerative changes in the LFJ cartilage, and showed that LFJ degeneration may be correlated with changes in the NP and PAF of the IVD.

## Supporting information

S1 Text The model assumed for data fitting in MRI mapping. (DOCX)

S1 Fig ROIs for the IVD and LFJ, and the Pfirrmann grade of IVD shown from grade I to grade IV. (DOCX)

## References

[1] Duan CY, Espinoza Orias AA, Shott S, An HS, Andersson GB, Hu JZ, et al In vivo measurement of the subchondral bone thickness of lumbar facet joint using magnetic resonance imaging. Osteoarthritis and cartilage / OARS, Osteoarthritis Research Society. 2011;19(1):96–102. PubMed Central PMCID: PMC3011863. 10.1016/j.joca.2010.10.015 PMC3011863 21034837

[2] Gellhorn AC, Katz JN, Suri P. Osteoarthritis of the spine: the facet joints. Nature reviews Rheumatology. 2013;9(4):216–24. PubMed Central PMCID: PMC4012322. doi: 10.1038/nrrheum.2012.199 23147891 10.1038/nrrheum.2012.199 PMC4012322

[3] Zhou Z, Jiang B, Zhou Z, Pan X, Sun H, Huang B, et al Intervertebral disk degeneration: T1rho MR imaging of human and animal models. Radiology. 2013;268(2):492–500. doi: 10.1148/radiol.13120874 23579049 10.1148/radiol.13120874

[4] Cao Y, Zhang Y, Yin X, Lu H, Hu J, Duan C. 3D visualization of the lumbar facet joint after degeneration using propagation phase contrast micro-tomography. Scientific reports. 2016;6:21838 PubMed Central PMCID: PMC4764819. doi: 10.1038/srep21838 26907889 10.1038/srep21838 PMC4764819

[5] Kalichman L, Hunter DJ. Lumbar facet joint osteoarthritis: a review. Seminars in arthritis and rheumatism. 2007;37(2):69–80. doi: 10.1016/j.semarthrit.2007.01.007 17379279 10.1016/j.semarthrit.2007.01.007

[6] Manchikanti L, Hirsch JA, Pampati V. Chronic low back pain of facet (zygapophysial) joint origin: is there a difference based on involvement of single or multiple spinal regions? Pain physician. 2003;6(4):399–405. 16871288

[7] Stelzeneder D, Messner A, Vlychou M, Welsch GH, Scheurecker G, Goed S, et al Quantitative in vivo MRI evaluation of lumbar facet joints and intervertebral discs using axial T2 mapping. European radiology. 2011;21(11):2388–95. doi: 10.1007/s00330-011-2198-z 21748388 10.1007/s00330-011-2198-z

[8] Blumenkrantz G, Majumdar S. Quantitative magnetic resonance imaging of articular cartilage in osteoarthritis. European cells & materials. 2007;13:76–86. 17506024 10.22203/ecm.v013a08

[9] Kijowski R, Chaudhary R. Quantitative magnetic resonance imaging of the articular cartilage of the knee joint. Magnetic resonance imaging clinics of North America. 2014;22(4):649–69. doi: 10.1016/j.mric.2014.07.005 25442027 10.1016/j.mric.2014.07.005

[10] Wang L, Regatte RR. Quantitative mapping of human cartilage at 3.0T: parallel changes in T(2), T(1)rho, and dGEMRIC. Academic radiology. 2014;21(4):463–71. PubMed Central PMCID: PMC3949430. doi: 10.1016/j.acra.2013.12.010 24594416 10.1016/j.acra.2013.12.010 PMC3949430

[11] Singh A, Haris M, Cai K, Kogan F, Hariharan H, Reddy R. High resolution T1rho mapping of in vivo human knee cartilage at 7T. PloS one. 2014;9(5):e97486 PubMed Central PMCID: PMC4022681. doi: 10.1371/journal.pone.0097486 24830386 10.1371/journal.pone.0097486 PMC4022681

[12] Hannila I, Lammentausta E, Tervonen O, Nieminen MT. The repeatability of T2 relaxation time measurement of human knee articular cartilage. Magma. 2015;28(6):547–53. doi: 10.1007/s10334-015-0494-3 26162930 10.1007/s10334-015-0494-3

[13] Kim HK, Horn P, Dardzinski BJ, Kim DH, Laor T. T2 Relaxation Time Mapping of the Cartilage Cap of Osteochondromas. Korean journal of radiology. 2016;17(1):159–65. PubMed Central PMCID: PMC4720804. doi: 10.3348/kjr.2016.17.1.159 26798229 10.3348/kjr.2016.17.1.159 PMC4720804

[14] Chen C, Huang M, Han Z, Shao L, Xie Y, Wu J, et al Quantitative T2 magnetic resonance imaging compared to morphological grading of the early cervical intervertebral disc degeneration: an evaluation approach in asymptomatic young adults. PloS one. 2014;9(2):e87856 PubMed Central PMCID: PMC3912130. doi: 10.1371/journal.pone.0087856 24498384 10.1371/journal.pone.0087856 PMC3912130

[15] Newbould RD, Miller SR, Toms LD, Swann P, Tielbeek JA, Gold GE, et al T2* measurement of the knee articular cartilage in osteoarthritis at 3T. Journal of magnetic resonance imaging: JMRI. 2012;35(6):1422–9. PubMed Central PMCID: PMC4351813. doi: 10.1002/jmri.23598 22314961 10.1002/jmri.23598 PMC4351813

[16] Bittersohl B, Hosalkar HS, Miese FR, Schibensky J, Konig DP, Herten M, et al Zonal T2* and T1Gd assessment of knee joint cartilage in various histological grades of cartilage degeneration: an observational in vitro study. BMJ open. 2015;5(2):e006895 PubMed Central PMCID: PMC4322206. doi: 10.1136/bmjopen-2014-006895 25667150 10.1136/bmjopen-2014-006895 PMC4322206

[17] Bittersohl B, Miese FR, Hosalkar HS, Herten M, Antoch G, Krauspe R, et al T2* mapping of hip joint cartilage in various histological grades of degeneration. Osteoarthritis and cartilage / OARS, Osteoarthritis Research Society. 2012;20(7):653–60. 10.1016/j.joca.2012.03.011 22469845

[18] Welsch GH, Trattnig S, Paternostro-Sluga T, Bohndorf K, Goed S, Stelzeneder D, et al Parametric T2 and T2* mapping techniques to visualize intervertebral disc degeneration in patients with low back pain: initial results on the clinical use of 3.0 Tesla MRI. Skeletal radiology. 2011;40(5):543–51. doi: 10.1007/s00256-010-1036-8 20878155 10.1007/s00256-010-1036-8

[19] Regatte RR, Akella SV, Lonner JH, Kneeland JB, Reddy R. T1rho relaxation mapping in human osteoarthritis (OA) cartilage: comparison of T1rho with T2. Journal of magnetic resonance imaging: JMRI. 2006;23(4):547–53. doi: 10.1002/jmri.20536 16523468 10.1002/jmri.20536

[20] Wang L, Chang G, Xu J, Vieira RL, Krasnokutsky S, Abramson S, et al T1rho MRI of menisci and cartilage in patients with osteoarthritis at 3 T. European journal of radiology. 2012;81(9):2329–36. PubMed Central PMCID: PMC3298732. 10.1016/j.ejrad.2011.07.017 PMC3298732 21908122

[21] Zhang X, Yang L, Gao F, Yuan Z, Lin X, Yao B, et al Comparison of T1rho and T2* Relaxation Mapping in Patients with Different Grades of Disc Degeneration at 3T MR. Medical science monitor: international medical journal of experimental and clinical research. 2015;21:1934–41. PubMed Central PMCID: PMC4501651. 26141783 10.12659/MSM.894406 PMC4501651

[22] Takashima H, Takebayashi T, Yoshimoto M, Terashima Y, Ida K, Shishido H, et al Investigation of intervertebral disc and facet joint in lumbar spondylolisthesis using T2 mapping. Magnetic resonance in medical sciences: MRMS: an official journal of Japan Society of Magnetic Resonance in Medicine. 2014;13(4):261–6. 10.2463/mrms.2013-0099 25345413

[23] Rodrigues LM, Oliveira LZ, Pinhal MA. Expression of heparanase isoforms in intervertebral discs classified according to Pfirrmann grading system for disc degeneration. Spine. 2013;38(13):1112–8. doi: 10.1097/BRS.0b013e3182894cf4 23370684 10.1097/BRS.0b013e3182894cf4

[24] Griffith JF, Wang YX, Antonio GE, Choi KC, Yu A, Ahuja AT, et al Modified Pfirrmann grading system for lumbar intervertebral disc degeneration. Spine. 2007;32(24):E708–12. doi: 10.1097/BRS.0b013e31815a59a0 18007231 10.1097/BRS.0b013e31815a59a0

[25] Rim DC. Quantitative Pfirrmann Disc Degeneration Grading System to Overcome the Limitation of Pfirrmann Disc Degeneration Grade. Korean Journal of Spine. 2016;13(1):1–8. PubMed Central PMCID: PMC4844654. doi: 10.14245/kjs.2016.13.1.1 27123023 10.14245/kjs.2016.13.1.1 PMC4844654

[26] Blumenkrantz G, Zuo J, Li X, Kornak J, Link TM, Majumdar S. In vivo 3.0-tesla magnetic resonance T1rho and T2 relaxation mapping in subjects with intervertebral disc degeneration and clinical symptoms. Magnetic resonance in medicine. 2010;63(5):1193–200. PubMed Central PMCID: PMC2988488. doi: 10.1002/mrm.22362 20432290 10.1002/mrm.22362 PMC2988488

[27] Borthakur A, Maurer PM, Fenty M, Wang C, Berger R, Yoder J, et al T1rho magnetic resonance imaging and discography pressure as novel biomarkers for disc degeneration and low back pain. Spine. 2011;36(25):2190–6. PubMed Central PMCID: PMC4002043. doi: 10.1097/BRS.0b013e31820287bf 21358489 10.1097/BRS.0b013e31820287bf PMC4002043

[28] Hoppe S, Quirbach S, Mamisch TC, Krause FG, Werlen S, Benneker LM. Axial T2 mapping in intervertebral discs: a new technique for assessment of intervertebral disc degeneration. European radiology. 2012;22(9):2013–9. doi: 10.1007/s00330-012-2448-8 22544293 10.1007/s00330-012-2448-8

[29] Marinelli NL, Haughton VM, Anderson PA. T2 relaxation times correlated with stage of lumbar intervertebral disk degeneration and patient age. AJNR American journal of neuroradiology. 2010;31(7):1278–82. doi: 10.3174/ajnr.A2080 20360340 10.3174/ajnr.A2080 PMC7965459

[30] Prasad AP, Nardo L, Schooler J, Joseph GB, Link TM. T(1)rho and T(2) relaxation times predict progression of knee osteoarthritis. Osteoarthritis and cartilage / OARS, Osteoarthritis Research Society. 2013;21(1):69–76. PubMed Central PMCID: PMC3596874. 10.1016/j.joca.2012.09.011 PMC3596874 23059757

[31] Bittersohl B, Hosalkar HS, Sondern M, Miese FR, Antoch G, Krauspe R, et al Spectrum of T2* values in knee joint cartilage at 3 T: a cross-sectional analysis in asymptomatic young adult volunteers. Skeletal radiology. 2014;43(4):443–52. doi: 10.1007/s00256-013-1806-1 24425347 10.1007/s00256-013-1806-1

[32] Kim T, Min BH, Yoon SH, Kim H, Park S, Lee HY, et al An in vitro comparative study of T2 and T2* mappings of human articular cartilage at 3-Tesla MRI using histology as the standard of reference. Skeletal radiology. 2014;43(7):947–54. doi: 10.1007/s00256-014-1872-z 24715200 10.1007/s00256-014-1872-z

[33] Kumar D, Souza RB, Singh J, Calixto NE, Nardo L, Link TM, et al Physical activity and spatial differences in medial knee T1rho and t2 relaxation times in knee osteoarthritis. The Journal of orthopaedic and sports physical therapy. 2014;44(12):964–72. PubMed Central PMCID: PMC4476033. doi: 10.2519/jospt.2014.5523 25353261 10.2519/jospt.2014.5523 PMC4476033

[34] Schooler J, Kumar D, Nardo L, McCulloch C, Li X, Link TM, et al Longitudinal evaluation of T1rho and T2 spatial distribution in osteoarthritic and healthy medial knee cartilage. Osteoarthritis and cartilage / OARS, Osteoarthritis Research Society. 2014;22(1):51–62. PubMed Central PMCID: PMC3934359. 10.1016/j.joca.2013.10.014 PMC3934359 24188868

[35] Hirose J, Nishioka H, Nakamura E, Oniki Y, Yamashita Y, Mizuta H. T1rho and T2 mapping of the proximal tibiofibular joint in relation to aging and cartilage degeneration. European journal of radiology. 2012;81(10):2776–82. doi: 10.1016/j.ejrad.2011.11.019 22153747 10.1016/j.ejrad.2011.11.019

[36] Souza RB, Kumar D, Calixto N, Singh J, Schooler J, Subburaj K, et al Response of knee cartilage T1rho and T2 relaxation times to in vivo mechanical loading in individuals with and without knee osteoarthritis. Osteoarthritis and cartilage / OARS, Osteoarthritis Research Society. 2014;22(10):1367–76. PubMed Central PMCID: PMC4184934. 10.1016/j.joca.2014.04.017 PMC4184934 24792208

[37] Fujiwara A, Tamai K, Yamato M, An HS, Yoshida H, Saotome K, et al The relationship between facet joint osteoarthritis and disc degeneration of the lumbar spine: an MRI study. European spine journal: official publication of the European Spine Society, the European Spinal Deformity Society, and the European Section of the Cervical Spine Research Society. 1999;8(5):396–401. PubMed Central PMCID: PMC3611192. 10.1007/s005860050193 PMC3611192 10552323

[38] Wang YX, Zhao F, Griffith JF, Mok GS, Leung JC, Ahuja AT, et al T1rho and T2 relaxation times for lumbar disc degeneration: an in vivo comparative study at 3.0-Tesla MRI. European radiology. 2013;23(1):228–34. doi: 10.1007/s00330-012-2591-2 22865227 10.1007/s00330-012-2591-2

