# Supplementary material for: Correlation study between facet joint cartilage and intervertebral discs in early lumbar vertebral degeneration using T2, T2* and T1ρ mapping
Source: PLoS One. 2017 Jun 1;12(6):e0178406. doi: 10.1371/journal.pone.0178406 (PMC5453520; doi:10.1371/journal.pone.0178406)
Supplement: S1 Text — (DOCX) [file pone.0178406.s001.docx]

**Supporting Information**

**S1 Text. The model assumed for data fitting in MRI mapping.**

A mono-exponential model, with the classical Levenberg–Marquardt algorithm (LMA), was used for data fitting in this study. In details, *S=S0*exp(-TE/T2)* was used for T2 mapping, *S=S0*exp(-TE/T2*)* was used for T2* mapping and *S=S0*exp(-TSL/T1ρ)* was used for T1ρ mapping. In the above equations, S0 represents reference signal intensity at TE=0ms or Spin lock time (TSL) = 0 ms, S is signal intensity at a given TE or TSL. The reason we chose this model was because this was the most suitable model which was determined by the principle of the MR scanning sequences for fitting T2, T2* and T1ρ maps.
