# Supplementary material for: Correlation study between facet joint cartilage and intervertebral discs in early lumbar vertebral degeneration using T2, T2* and T1ρ mapping
Source: PLoS One. 2017 Jun 1;12(6):e0178406. doi: 10.1371/journal.pone.0178406 (PMC5453520; doi:10.1371/journal.pone.0178406)
Supplement: S1 Fig — (DOCX) [file pone.0178406.s002.docx]

**Supporting Information**

**
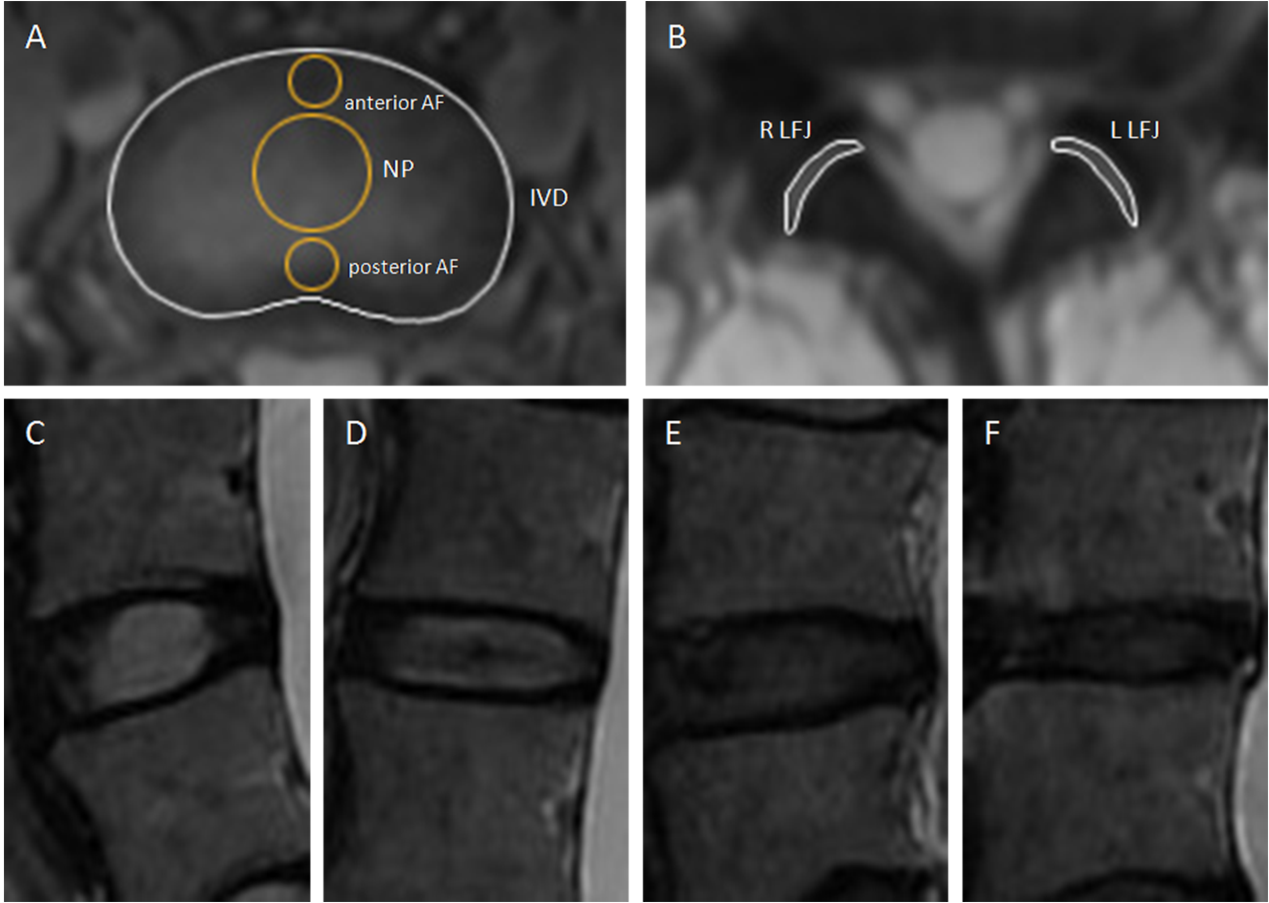
**

**S1 Fig. ROIs for the IVD and LFJ, and the Pfirrmann grade of IVD shown for grade I to grade IV.** The regions of interest (ROIs) for the IVD (Fig 1A) and lumbar facet joint cartilage (Fig 1B) were drawn on the first echo image of mapping, and the Pfirrmann grade of IVD shown for grade I to grade IV (Fig 1C~F) in sagittal FSE T2WI. Once the ROIs had been drawn, a same shape would be copied and pasted into the T2 /T2*/T1ρ colored map to measure values. For the ROIs of IVD in Fig 1A, a large circular ROI was selected for NP (about 60 percent of the mid-sagittal disc diameter) and two equal small circular ROIs were drawn for anterior and posterior AF. Fig 1B shows the ROIs of lumbar facet joints cartilage area (left and right).
